# Editorial: Diagnosis and therapy pediatric hematological malignancies: recent progress

**DOI:** 10.3389/fped.2023.1303561

**Published:** 2023-10-26

**Authors:** Joanna Zawitkowska, Monika Lejman, Katarzyna Derwich

**Affiliations:** ^1^ Department of Pediatric Hematology, Oncology and Transplantology, Medical University of Lublin, Lublin, Poland; ^2^ Independent Laboratory of Genetic Diagnostics, Medical University of Lublin, Lublin, Poland; ^3^ Department of Pediatric Oncology, Hematology and Transplantology, Poznan University of Medical Sciences, Poznan, Poland

**Keywords:** hematological malignancies, children, diagnosis, therapy, molecular methods

**Editorial on the Research Topic** Diagnosis and therapy pediatric hematological malignancies: recent progress

Hematological malignancies are the most frequent cancer in the pediatric population. Leukemia represents approximately 28% of cancer diagnosis (acute lymphoblastic leukemia being the most common type of cancer), while lymphomas account for 9% (third after brain tumors) (1). In recent decades, the overall survival (OS) rate for childhood hematological malignancies has increased to 80%–90% in high-income countries. This was possible due to stratified risk classification (minimal residual disease, MRD), intensive chemotherapy, steroid therapy, and radiotherapy (2, 3). However, difficulties still remain, such as treatment-related mortality and relapse/refractory disease (4). Further increasing the dosage of conventional chemotherapy does not improve the outcome and it intensifies complications. Innovative approaches to genetic abnormalities in childhood hematological cancer led to the introduction of new therapy options: molecular target therapy, immunotherapy, and chimeric antigen receptor (CAR) T cells (5–8).

Liu et al. investigated the genomic signatures (by whole exome sequencing) and prognosis of advanced-stage T cell lymphoblastic lymphoma (T-LBL) in 35 children. The authors concluded that USP34 overexpression and the PI3K-Akt signaling pathway are connected with T-LBL development and progression, but anticancer therapies targeting the USP34 gene or the PI3K-Akt signaling pathway may be ineffective for children with refractory relapsed T-LBL (Liu et al.). Ochoa-Fernández et al. reported the outcomes of 39 patients diagnosed with infant leukemia from 1990 to 2020. The main risk factors that affected survival in this study were an age younger than 6 months and a poor response to induction therapy. The authors showed that KMT2A-r was not associated with poorer outcomes in either ALL or AML, probably due to the small cohort of patients. The 5-year OS and 5-year even free survival (EFS) were analyzed for two periods (1990–2005 and 2006–2020). For the ALL patients in the first period, the 5-year EFS and 5-year OS were the same at 30% and in the second period were 41.2% and 44.2%, respectively. For AML patients in the first period, the 5-year EFS and 5-year OS were the same at 66.7% and in the second period were 62.5% and 75%, respectively. There was no statistically significant difference in EFS or OS between these periods. These results show that new therapeutic approaches could improve outcomes in the infant population (Ochoa-Fernández et al.). Very interesting observations were presented by Li et al. regarding the clinical characteristics of pediatric patients with hematological diseases with novel coronavirus infection in the outpatient and emergency department of the Seventh Affiliated Hospital of Sun Yat-sen University. The study group included 20 children who were divided into two groups: group A consisted of children with hematologic neoplastic diseases treated with paxlovid and Group B of patients with hematological malignancies who did not receive paxlovid treatment. The authors evaluated the safety and efficacy of paxlovid treatment in the analyzed study group. The authors concluded that paxlovid can shorten the virus clearance time and reduce the risk of developing severe disease. In this study, no serious adverse effects of paxlovid were observed. However, due to the lack of safety and pharmacokinetic studies, paxlovid is not currently recommended (Li et al.).

Lv et al. presented the outcomes of high-throughput drug sensitivity screening (HDS) in children with rel/ref AML. The authors analyzed 37 children with rel/ref AML who received HDS. A total of 24 patients (64.9%) had adverse cytogenetics, and two children had rel/ref AML with central nervous system infiltration. The 3-year overall survival (OS) and EFS rates were 45.9% and 43.2%, respectively. The main cause of death was infection during neutropenia. The results of this study suggest that the HDS-guided chemotherapy regimen is associated with a high bone marrow remission rate and a good safety profile in childhood rel/ref AML, and therefore, may be a good bridging treatment for stem cell transplantation. However, a prospective study for a large patient group is necessary (Lv et al.).

In summary, the diagnosis of hematologic malignancies, patient management, supportive care, and assessment of treatment response have improved significantly. The development of molecular research has led to the genetic characterization of childhood cancers, resulting in molecularly targeted agents and immunotherapies.

## References

[1] Siegel RL Miller KD Fuchs HE Jemal A. Cancer statistics, 2021. CA Cancer J Clin. (2021) 71:7–33. 10.3322/caac.21654 33433946

[2] Inaba H Pui CH. Advances in the diagnosis and treatment of pediatric acute lymphoblastic leukemia. J Clin Med. (2021) 10(9):1926. 10.3390/jcm10091926 33946897 PMC8124693

[3] Cairo MS Beishuizen A. Childhood, adolescent and young adult non-Hodgkin lymphoma: current perspectives. Br J Haematol. (2019) 185(6):1021–42. 10.1111/bjh.15764 30729513 PMC6897376

[4] Inaba H Pei D Wolf J Howard SC Hayden RT Go M Infection-related complications during treatment for childhood acute lymphoblastic leukemia. Ann Oncol. (2017) 28:386–92. 10.1093/annonc/mdw557 28426102 PMC5834143

[5] Locatelli F Zugmaier G Rizzari C Morris JD Gruhn B Klingebiel T Effect of blinatumomab vs. chemotherapy on event-free survival among children with high-risk first-relapse B-cell acute lymphoblastic leukemia: a randomized clinical trial. JAMA. (2021) 325:843–54. 10.1001/jama.2021.0987 33651091 PMC7926287

[6] Majzner RG Mackall CL. Tumor antigen escape from CAR T-cell therapy. Cancer Discov. (2018) 8:1219–26. 10.1158/2159-8290 30135176

[7] Neelapu S Adkins S Ansell SM Brody J Cairo MS Friedberg JW Society for immunotherapy of cancer (SITC) clinical practice guideline on immunotherapy for the treatment of lymphoma. J Immunother Cancer. (2020) 8:e001235. 10.1136/jitc-2020-001235 33361336 PMC7768967

[8] Ribeiro ML Reyes-Garau D Armengol M Fernández-Serrano M Roué G. Recent advances in the targeting of epigenetic regulators in B-cell non-Hodgkin lymphoma. Front Genet. (2019) 10:986. 10.3389/fgene.2019.00986 31681423 PMC6807552

